# Automated quantification of bioluminescence images

**DOI:** 10.1038/s41467-018-06288-w

**Published:** 2018-10-15

**Authors:** Alexander D. Klose, Neal Paragas

**Affiliations:** 1 InVivo Analytics, New York, NY USA; 2 University of Washington, Seattle, WA USA

## Abstract

We developed a computer-aided analysis tool for quantitatively determining bioluminescent reporter distributions inside small animals. The core innovations are a body-fitting animal shuttle and a statistical mouse atlas, both of which are spatially aligned and scaled according to the animal’s weight, and hence provide data congruency across animals of varying size and pose. In conjunction with a multispectral bioluminescence tomography technique capitalizing on the spatial framework of the shuttle, the in vivo biodistribution of luminescent reporters can rapidly be calculated and, thus, enables operator-independent and computer-driven data analysis. We demonstrate its functionality by quantitatively monitoring a bacterial infection, where the bacterial organ burden was determined and validated with the established serial-plating method. In addition, the statistical mouse atlas was validated and compared to existing techniques providing an anatomical reference. The proposed data analysis tool promises to increase data throughput and data reproducibility and accelerate human disease modeling in mice.

## Abstract (web summary)

Analysis of bioluminescence images of bacterial distributions in living animals is mostly manual and semiquantitative. Here, the authors present an analysis platform featuring an animal mold, a probabilistic organ atlas, and a mirror gantry to perform automatic in vivo bioluminescence quantification.

## Introduction

Bioluminescence imaging (BLi) is an established small animal imaging modality for in vivo monitoring of bioluminescent reporter systems^1–8^. BLi is, however, mostly limited to imaging of diffuse light intensity distributions on the tissue surface, which are caused by light scattering and partial light absorption of tissue. The source of light emission, e.g., the spatial distribution of luminescent bacteria or the volume and location of cancerous tumors and metastases, is not directly accessible for quantitation. Therefore, investigators are restricted to either invasive methods such as serial plating of bacteria or excising a tumor using a study endpoint, or to semiquantitative methods such as the assessment of relative fold-changes in user-defined regions-of-interests (ROI) of bioluminescence images.

We chose bacterial infections as an experimental model for our feasibility study because they are an exemplary case for demonstrating the limitations of BLi in preclinical research and drug development. Bacterial infections impose a costly health burden worldwide, which is compounded by the alarming increase of multidrug resistant (MDR) gram-negative bacteria^9^, and many of these infections are in the urogenital tract. An important tool for the development of novel antibiotics to combat MDR *Escherichia coli* is in vivo imaging of bacterial infections in small animals using bioluminescent bacteria^10–12^. Quantitative data analysis of luminescent bacteria inside tissue is, however, challenging because images of light intensity distributions on the tissue surface do not directly provide the absolute in vivo bacterial organ burden^13,14^.

**Fig. 1:**
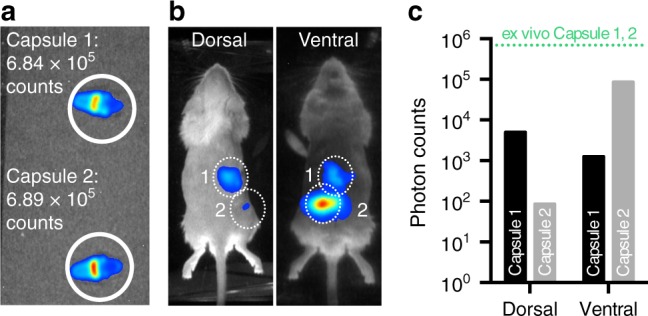
Challenges in bioluminescence imaging. Planar in vivo BLi is qualitative or semiquantitative. **a** Capsules 1 and 2 with equivalent CFUs (10^8^) of lux-bacteria. **b** In vivo BLi of capsules implanted at different locations inside an animal yields **c** different light intensities (photon count) at the tissue surface dependent on capsule location and camera view

**Fig. 2:**
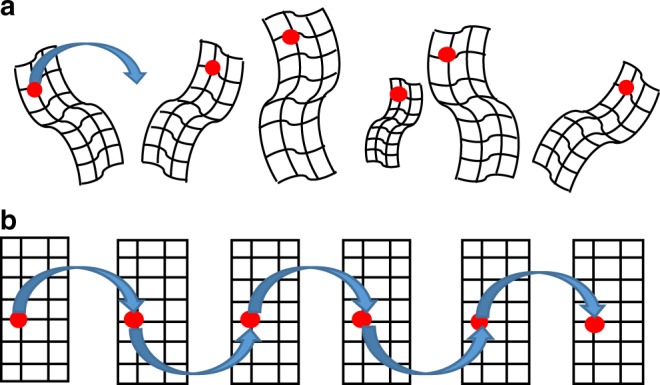
Challenges for automated data analysis. Missing spatial data point congruency between animals prohibits automated data analysis. **a** Inherent data point relation between animals is not available because of different animal sizes, positions, and poses. Manual ROI delineation is required for data analysis. **b** Data congruency between animals is enabled via the BCAM. Automated ROI delineation and data analysis becomes feasible

Current data analysis of bioluminescence images relies on the following assumptions. First, the measured light intensity at the animal’s body surface (photons s^−1^ mm^−2^) is assumed to linearly correlate with its unknown luminescence source inside tissue^10^. Light is, however, strongly attenuated by tissue and the measured intensities are nonlinearly dependent on (i) the spatial location of the bioluminescence source, (ii) the heterogeneous optical tissue properties, (iii) the animal’s size, position, and shape, and (iv) the imaging view and relative position of the animal to the optical camera (Fig. 1a–c)^15,16^. Second, the absence of a defined geometric framework addressing the varying animal size and pose and the requirement for manually drawing ROIs in collected bioluminescence images prohibits automated image analysis of entire animal cohorts and studies (Fig. 2a). An inherent data congruency is missing that could automatically relate individual image data points across all animals. Quantitative and automated analysis requires a tool that (i) determines the actual luminescence source inside tissue and (ii) enables data congruency across all images.

Previous attempts for achieving quantitative imaging results have been made with bioluminescence tomography (BLt)^17–26^. BLt directly reconstructs the photon emission density (photons s^−1^ mm^−3^) of a luminescent reporter inside tissue by using an image reconstruction algorithm and spectral light intensity measurements at the tissue surface. The reconstruction algorithm iteratively updates the unknown luminescence source distribution by comparing light intensity predictions to the measured intensities. The predictions are determined with a light propagation model that requires knowledge about the optical tissue properties and the surface geometry of the animal. The light propagation model can be solved numerically with computationally expensive finite difference (FD) or finite element (FE) methods^15,16,27^. Because the numerical solutions depend on the animal’s geometry, which is also different for each individual animal, they need to be obtained for each image reconstruction task and, therefore, it imposes a practical limitation in longitudinal studies with many animals. Once the source distribution has been reconstructed, it can directly be correlated to the size and location of the unknown luminescent reporter, such as the bacterial density distribution. For that purpose, the photon emission density distribution is linearly translated, for example, into a spatial map of colony forming units (CFU) of bacteria by using a known CFU and light emission density obtained from an independent calibration experiment.

The animal’s geometry for the purpose of light propagation modeling can be obtained by using either a structured light technique for capturing the tissue surface (LivingImage, PerkinElmer)^22^ or an X-ray computed tomography (CT) scan^28^. The first approach projects a structured light pattern by a parallel laser beam under a defined angle onto the animal’s surface. A registration software recovers the depth information along the object-to-camera axis. This method captures a single view of the animal that is directly exposed to the camera and, hence, leads to a shadow cast resulting in an incomplete surface map. The second approach utilizes a CT scan of the animal followed by an image segmentation of its body surface. Although this technique yields the complete surface geometry, it still relies on relatively expensive and complex hardware.

Automated biodistribution analysis of entire studies requires: (i) the animal’s anatomy for determining the luminescent reporter expression or uptake in each organ and (ii) a standardized spatial framework across all animals of different size and pose for enabling data congruency. Providing an anatomical reference has partially been addressed by either aligning a digital mouse atlas (e.g., Digimouse^29^ and MOBY mouse phantom^30^) to the silhouettes of mice^21,31–38^ or by co-registering the BLt reconstructions to an organ-segmented CT scan^28^. Successful organ segmentation depends, however, upon a human operator to manually or semiautomatically segment images. While CT easily enables the delineation of high contrast organs such as skeleton and lungs, a complete body segmentation still relies on a contrast agent due to the poor soft-tissue contrast of most organs. Therefore, routine imaging might not only be impractical but also prohibitively expensive in longitudinal studies with multiple arms and large cohorts. On the other hand, the coalignment of BLt maps to a digital mouse atlas can be accomplished either manually or by the aid of additional registration algorithms and fiducial marks^21,32^. The latter technique, for example, uses an atlas deformation model, which warps a mouse atlas to the registered body surface of each animal. Atlas deformation models have been studied extensively and have yielded promising results; however, they remain technically cumbersome and computationally demanding with relatively long processing times^33,34^.

Addressing the mentioned limitations and challenges, we have developed a data analysis tool, InVivoPLOT, that enables quantitative and operator-independent in vivo monitoring of bioluminescent reporter distributions. We demonstrate its feasibility on a bacterial infection model, where it calculates the in vivo bacterial density distribution [CFU mm^−3^] inside the animal and automatically determines the bacterial organ load. InVivoPLOT differentiates itself from current methods: (i) it can quantitate in vivo bioluminescent targets across different animals and time points, (ii) automatically registers it to an anatomical reference, and (iii) performs all image data analysis without any operator bias.

## Results

### Automated data analysis of bioluminescence images

InVivoPLOT is comprised of several innovations: a body-conforming animal mold (BCAM) for providing a defined spatial geometry across animals of different body weights; an organ probability map (OPM) constituting a statistical mouse atlas for different strains and sex; a cloud-based image reconstruction algorithm for BLt^16,24,27^; and a plug-in unit with mirror gantry for bioluminescence imaging systems. InVivoPLOT transforms multispectral BLi images of animals inside the BCAM into 3D spatial maps of bioluminescence source density distributions. The calculated 3D maps are coregistered to the OPM, which automatically extracts ROIs based on the animal’s anatomy. The BCAM ensures data congruency across animals with different size and, hence, enables automated image data analysis without the need for a human operator and manual organ ROI delineation. We validated InVivoPLOT in a urinary tract infection (UTI) model that demonstrated feasibility for mapping the spatial bacterial burden (CFU mm^−3^) as bacteria ascend from a superficial organ (bladder) to a deep-set organ (kidney) via the ureters in a living mouse^39^.

### Data congruency enabled by body-conforming animal mold

**Fig. 3:**
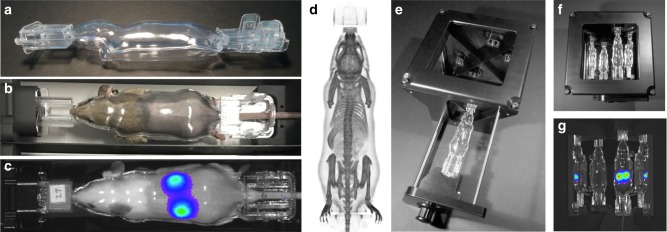
Body-conforming animal mold and mirror gantry. BCAM provides known surface geometry for BLt and enables atlas coregistration. Mirror gantry provides multiview image of mouse for BLt. **a** Optically transparent BCAM consisting of top and bottom shell. **b** BCAM with combined gas anesthesia supply and gas scavenging port holds animal in an immobilized and geometrically defined position and pose. **c** Bioluminescence image of animal with lux-bacteria in kidneys. **d** X-ray projection of mouse inside BCAM. **e** Mirror gantry for multiview imaging. **f** Photograph showing direct (dorsal) and reflected BCAM (ventral, left, right) views. **g** Multiview camera image of luminescent mouse shows dorsal, ventral, left, and right view of bioluminescent kidneys

InVivoPLOT’s core innovation is the BCAM (Fig. 3a), an optically transparent animal shuttle consisting of two rigid clam shells, in the form of an average body shape of a mouse. The BCAM holds the mouse body in a fixed and confined pose without exposing the animal to any physiological stress or respiratory constrains (Fig. 3b–d). It is resistant to common disinfectants and sterilization techniques to permit repeated use. The BCAM design serves four main functions. First, the fixed animal pose yields data congruency across mice of different size and, therefore, enables automated data analysis (Fig. 2b). Second, the BCAM provides a defined geometry (Supplementary Movie 1), which is required for solving the light propagation model as part of the BLt algorithm. Most importantly though, the light propagation model does not need to be solved during the image reconstruction process, which ultimately saves computation time. Its solutions have been obtained prior to image reconstruction because the surface geometry of animals inside the BCAM was already known. Third, the BCAM enables the direct alignment of a digital mouse atlas without the need for an atlas deformation model morphing to each individual animal’s surface geometry. Last, the BCAM permits automatic and repetitive coregistration between different imaging modalities (e.g., CT and optical) without changing the pose or position of the animal.

The BCAM volume is also scaled according to the animal’s body weight while ensuring the required body fit (Supplementary Movie 1, Supplementary Figs. 1, 2). This assumption is based on the almost uniform tissue density of 1.03–1.06 g cm^−3^ for animals smaller than 40 g^40^. The body length also scales linearly for weights smaller than 38 g, while maintaining the same body to fat ratio^38,40^. Hence, we have developed a total of 22 BCAMs from 17 to 38 g with weight increments of 1 g (Supplementary Fig. 1), which is covering the weight range of most mouse strains^41^. The relatively small weight increments ensure that the surface distance between consecutive BCAM sizes changes only by 100–170 µm along the dorsal–ventral axis and by 450–700 µm along the anterior–posterior axis. We validated the goodness of fit of the animal surface (skin/fur) with the interior BCAM surface with CT scans by visually examining the coronal, sagittal, and trans-axial CT image sections (Supplementary Fig. 2).

The injection molded BCAM is made of optically clear polycarbonate (Makrolon (Bayer), 89% transmittance, refractive index *n* = 1.58) providing optical transmittance for bioluminescence light (Fig. 3c). The critical angle of internal reflection at the BCAM-to-air interface is approximately 40°. Photons exiting the BCAM surface under a larger angle will be totally reflected while partially entering the animal tissue. We found, however, that the contribution of light that is internally reflected does not significantly impact the total light intensity distribution measured by the optical camera. We demonstrated the impact of the BCAM on the light reflection by performing a comparative imaging experiment, where the top shell of the BCAM was in place or removed. Two mice (27 and 29 g, respectively) with kidney-specific luciferase expression were placed into the weight-designated BCAMs, top shell was either in place or removed, and bioluminescence images were acquired with an IVIS Spectrum (PerkinElmer). The tissue surface was directly exposed to the camera when the top shell was removed. Comparing the results of both experimental conditions demonstrated that the BCAM showed no significant impact on the light intensity distribution at the tissue surface (Supplementary Fig. 3).

### Mirror gantry for multiview image acquisition

The mirror gantry facilitates simultaneous 360° multiview imaging of the BCAM for performing multispectral BLt. The gantry consists of an aluminum housing, two broadband metallic mirrors (surface coated, aluminum, reflection > 90% at visible wavelengths), and a BCAM adapter with gas anesthesia port (Fig. 3e–g). A system-specific adapter plate positions the gantry at a fixed location inside the imaging system. The adapter plate is manufacturer-specific and makes the gantry compatible for different optical imaging systems. As part of this study, we developed adapters for both a PhotonImager (BiospaceLab) and an IVIS Spectrum (PerkinElmer). The top side of the gantry has a clear window, which directly exposes the BCAM to the optical camera of the imaging system. Both mirrors meet at a 90° angle, while the BCAM is placed above one mirror. Such configuration enables simultaneous imaging of four views of the BCAM: the top view (dorsal side) directly exposed to the camera, both lateral views (left and right sides) with a single mirror reflection, and the bottom view (ventral side) with a double-reflection. Hence, each view captures a 90° segment of the full BCAM surface. Since the BCAM is locked into the same spatial position inside the gantry, coregistration of light intensities across BCAMs of different animal weights becomes possible. It also ensures a consistent and reproducible cross-comparison of data sets, either for high-throughput BLi or 3D BLt image reconstruction.

### Statistical mouse atlas as anatomical reference

**Fig. 4:**
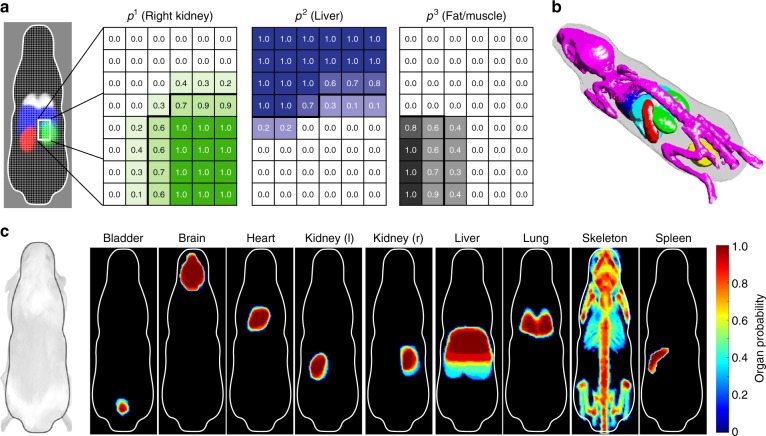
Organ probability map. OPM enables automated ROI delineation. **a** Schematic of OPM showing organ probabilities *p* of right kidney, liver, and connective tissue. Organ boundary is highlighted-black. **b** Surface rendering of male C57BL/6 OPM with probability threshold of 40%: BCAM (gray), bladder (yellow), brain (not visible), heart (red), kidneys (green), liver (light blue), lung (blue), skeleton (purple), and spleen (red). **c** Maximum intensity projection of OPM (coronal view) showing the maximum probabilities of bladder, brain, heart, left and right kidneys, liver, lung, skeleton, and spleen (BCAM boundary: white outline)

**Fig. 5:**
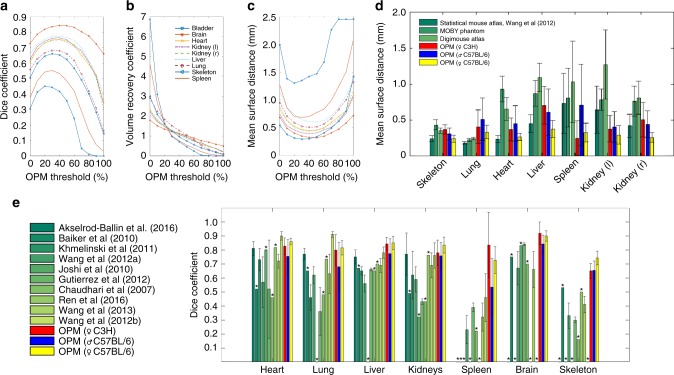
Validation of BCAM and OPM. The anatomical registration accuracy of the female C3H, male C57BL/6, and female C57BL/6 OPMs has been validated for major organs using different registration metrics. **a** Dice coefficient, **b** volume recovery coefficient (VRC), and **c** mean surface distance (MSD) of male C57BL/6 mice for different probability thresholds using an expert data set of segmented CT scans (*n* *=* 10). **d** MSD of OPMs (40% probability threshold) compared to results obtained by Wang et al.^42^. **e** Dice coefficients of OPMs (40% probability threshold) in comparison to different anatomical registration studies. These methods include: a multi-atlas registration for BLt (Ren et al. 2016^48^); deformable mouse atlas registration (Gutierrez et al. 2012^47^; Joshi et al. 2010^34^; Chaudhari et al. 2007^21^); multimodal organ segmentation and registration to BLi (Akselrod-Ballin et al. 2016^45^); statistical mouse atlas registration (Wang et al. 2012(a)^36^, Wang et al. 2012(b)^42^, and Wang et al. 2013^44^); automated organ segmentationusing atlas-based registration (Baiker et al. 2010^37^); and articulated atlas registration for BLi (Khmelinskii et al. 2011^46^). ^*^No data available

The OPM is the other core component of InVivoPLOT and constitutes a statistical mouse atlas (Fig. 4a). The atlas is intrinsically coaligned to the BCAM shape (Fig. 4b, c). The atlas serves two main functions. First, it instantaneously provides an anatomical reference for a specific mouse strain and sex without the need for additional body surface registration methods, manual atlas alignment, or atlas deformation models for morphing it to the animal’s body shape. Second, the OPM is built from multiple organ-segmented CT scans and, thus, also addresses: (i) the biological variation across different animals and (ii) the repeat placement of the same animal into the BCAM at different time points. Prior studies have shown that a multisubject animal atlas promises to be a more realistic representation of an expected organ distribution than an atlas of a single-subject mouse atlas (Fig. 5d)^38,42^.

An OPM for the purposes of this feasibility study has been built from a cohort of female C3H mice (*n* = 11) (Figs. 4c, 5d, e, Supplementary Figs. 4–12, and Supplementary Movie 2). The OPM was constructed from manual organ segmentations of contrast-enhanced CT scans including a total of nine organs: skeleton, brain, lung, liver, kidneys, heart, bladder, spleen, and eyes. In addition, a probability map of the BCAM was built from the same data set of CT scans. The spatial BCAM probability distribution also correlates with the body shape of the registered animals.

The OPM can be scaled via a rigid body transform to the animal’s body size defined by the weight designation of the BCAM and facilitated through the fixed spatial geometry of the BCAM (Fig. 3a, Supplementary Fig. 1). Therefore, the OPM was linearly scaled to fit the range of BCAMs between 17 and 38 g (Supplementary Fig. 1). Depending on the scaling factor for each BCAM size, the OPM has a voxel resolution between 0.44 and 0.58 mm. In addition to the female C3H OPM, we also built an OPM of female C57BL/6 mice (*n* = 9) to compare two different strains of same sex, and an OPM of male C57BL/6 mice (*n* = 11) to compare both sex of the same strain.

### Validation of statistical mouse atlas

The anatomical registration accuracy of the OPMs was validated with expert data sets of organ-segmented CT scans of female C3H (*n* = 9, 24 g), female C57BL/6 (*n* = 9, 20 g), and male C57BL/6 (*n* = 10, 17–31g) mice by calculating the Dice coefficients^43^, the volume recovery coefficients (VRC), the generalized mean surface distance (MSD) or average surface distance^44^, and the directional MSD (dMSD)^42^ for each segmented CT scan. The data in Table 1 are the mean and standard deviations of all metrics obtained from the entire set of CT scans.

**Table 1:** Dice coefficient, VRC, MSD, and dMSD

	Bladder		Brain		Heart		Kidney L		Kidney R		Liver		Lung		Skeleton		Spleen	
	Mean	Std	Mean	Std	Mean	Std	Mean	Std	Mean	Std	Mean	Std	Mean	Std	Mean	Std	Mean	Std
*C3H (female)*																		
Dice	0.582	0.167	0.918	0.081	0.827	0.059	0.804	0.082	0.751	0.102	0.843	0.047	0.799	0.11	0.65	0.053	0.834	0.234
VRC	1.52	1.71	1.03	0.035	1.07	0.214	1.07	0.197	1.03	0.171	1.11	0.233	1.08	0.334	0.956	0.12	0.941	0.128
MSD [mm]	0.593	0.274	0.198	0.202	0.366	0.157	0.376	0.184	0.502	0.24	0.701	0.261	0.403	0.233	0.367	0.066	0.242	0.352
dMSD [mm]	0.547	0.295	0.203	0.205	0.365	0.149	0.391	0.192	0.505	0.246	0.809	0.53	0.387	0.218	0.286	0.0826	0.196	0.292
*C57 (male)*																		
Dice	0.417	0.31	0.843	0.062	0.754	0.119	0.764	0.095	0.751	0.9	0.774	0.106	0.68	0.171	0.653	0.067	0.534	0.204
VRC	0.71	0.372	1.12	0.234	1.06	0.155	1.14	0.173	1.13	0.184	1.07	0.147	1.28	0.578	0.968	0.127	0.766	0.218
MSD [mm]	1.42	1.62	0.331	0.146	0.451	0.246	0.4	0.216	0.439	0.187	0.607	0.322	0.509	0.294	0.306	0.084	0.704	0.568
dMSD [mm]	1.32	1.71	0.372	0.208	0.481	0.277	0.446	0.257	0.513	0.219	0.644	0.431	0.613	0.483	0.272	0.082	0.504	0.317
*C57 (female)*																		
Dice	0.754	0.09	0.899	0.038	0.86	0.023	0.827	0.074	0.842	0.04	0.85	0.047	0.814	0.051	0.743	0.049	0.727	0.095
VRC	1.25	0.377	1.07	0.058	1.08	0.131	1.09	0.111	1.07	0.131	1.11	0.186	1.14	0.213	1.08	0.14	1.04	0.127
MSD [mm]	0.499	0.187	0.218	0.089	0.264	0.05	0.289	0.132	0.256	0.069	0.373	0.12	0.331	0.097	0.236	0.051	0.323	0.131
dMSD [mm]	0.569	0.262	0.229	0.093	0.277	0.064	0.305	0.143	0.27	0.077	0.399	0.173	0.349	0.139	0.229	0.056	0.321	0.139

Registration metrics have been calculated for the female C3H, male C57BL/6, and female C57BL/6 OPMs using a 40% probability threshold of the OPMs

First, we investigated the impact of different probability thresholds (*p* = 0.01–1.0) of the OPM on the anatomical registration accuracy (Fig. 5a–c). The largest Dice coefficient, a VRC close to 1, and the smallest MSD could be found for organ probability thresholds between 30% (*p* = 0.3) and 50% (*p* = 0.5). For example, a threshold of 40% (*p* = 0.4) can be interpreted as the value that at least four out of ten animals shared the same organ voxel.

Next, we calculated the Dice, VRC, MSD, and dMSD for all three OPMs using a 40% (*p* = 0.4) probability threshold (Table 1). Excluding the bladder, we obtained Dice coefficients between 0.53 ± 0.2 for the spleen and 0.91 ± 0.08 for the brain; and MSDs between 0.7 ± 0.56 mm for the spleen and 0.19 ± 0.2 mm for the brain. The MSD was compared to results reported by Wang et al.^42^ who were using a deformable atlas registration method applied to a multisubject statistical mouse atlas and single-subject mouse atlases, i.e., the MOBY phantom^30^ and the Digimouse^29^ (Fig. 5d). We compared the Dice coefficients of all OPMs to the Dice obtained from different anatomical registration methods^21,34,36,37,42,44–48^, and found an improvement in anatomical registration accuracy for most organs (Fig. 5e).

Last, we demonstrated that an OPM can precisely be scaled to fit animals with varying body weight and corresponding BCAM size. The 24 g BCAM constituted the standard normal with an OPM scale factor of 1.0, whereas the OPM needed to be geometrically scaled for animals with different weights to fit their associated BCAM sizes. The spatially isotropic scale factors had a range between 0.89 and 1.16 (17–38 g BCAM size). We validated the accurate coregistration of different BCAM sizes (*n* = 10) between 17 and 31 g to the probability map of the BCAM and obtained a mean Dice of 0.88 ± 0.03 and mean VRC of 1.08 ± 0.04 for the entire set of BCAMs. We compared those results to a Dice of 0.84 ± 0.05 and a VRC of 1.15 ± 0.05 of a corresponding registration study using only 20g-size BCAMs (*n* = 9). The Dice coefficients of the scaled OPM (male C57BL/6, 17–31 g) were between 0.53 ± 0.2 (spleen) and 0.84 ± 0.06 (brain) and slightly smaller than the Dice 0.72 ± 0.09 and 0.89 ± 0.03 of the corresponding organs of the OPM with fixed BCAM size (female C57BL/6, 20 g) (Table 1).

### Bioluminescence tomographic reconstruction

InVivoPLOT’s multispectral BLt method employs an image reconstruction algorithm for calculating the 3D bioluminescence source distribution inside the animal. It is based on an expectation-maximization (EM) method, where the projection matrix for each BCAM size has been constructed by a light propagation model. The light propagation model calculated each matrix element for a given light source location inside the animal and given detector point on the animal’s surface. The model is based on a FD implementation of the simplified spherical harmonics (SP_3_) equations and its boundary conditions^27^. The boundary conditions take the refractive index mismatch between the medium-to-air interface into account. Because the SP_3_ equations with boundary conditions are a high-order approximation to the radiative transfer equation for light, they overcome the limitations of low-order approximations (e.g., diffusion model) generally being used in BLt^15,18,49,50^. In contrast to the diffusion model, the SP_3_ model can be applied to the entire bioluminescence spectrum, including at wavelengths <620 nm where strong light absorption goes beyond the diffusion limit.

The projection matrix could be precalculated, which was only made possible by the known surface geometry of the BCAM. Therefore, InVivoPLOT could achieve rapid whole-body image reconstructions in less than 60 s. In comparison, the calculation of the projection matrix with more than 2.8 × 10^9^ matrix elements took ~125 h computation time on eight cores of two 2.7 GHz Intel Xeon processors. Without the utilization of the BCAM geometry for light propagation modeling, the BLt algorithm would have had to compute the projection matrix for every individual image reconstruction task. A repetitive matrix calculation would have widely exceeded any practical consideration for whole-body BLt.

### Validation of quantitative BLt approach

**Fig. 6:**
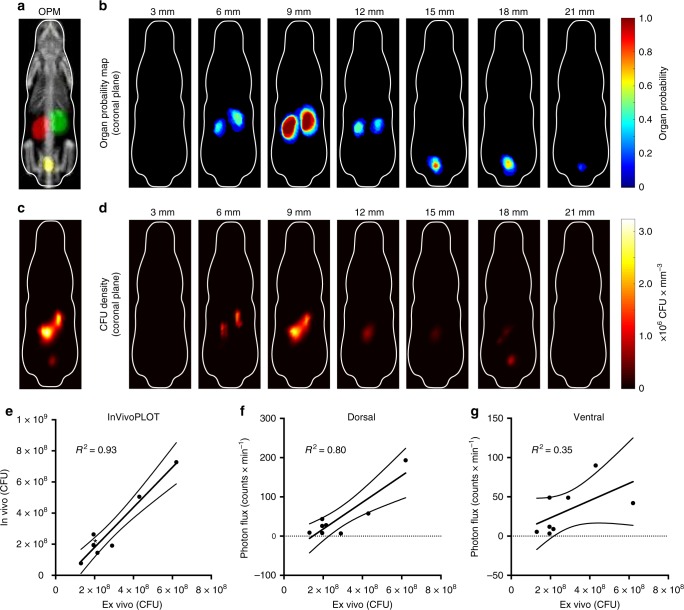
BLt reconstruction of bacterial infection with OPM coregistration. Spatial distribution of lux-bacteria was reconstructed in a UTI model of female C3H/HeJ mice (*n* = 8, 24 g). **a** Maximum intensity projection of kidney, bladder, and skeleton OPM and schematic outline of BCAM as reference. **b** Coronal slices of kidney and bladder OPM in 3 mm increments from the dorsal to ventral side. **c** Maximum intensity projection and **d** aligned coronal sections from reconstructed bacterial density distribution of mouse with pyelonephritis. **e** Calculated total in vivo CFU count of kidney using BLt vs total ex vivo CFU count of harvested kidneys. The Pearson correlation coefficient is *R*^2^ = 0.93. **f**, **g** Measured total light intensities of kidney ROI on animal surface using BLi vs total ex vivo CFU count of harvested kidneys quantified by serial plating. **f** Plot corresponding to dorsal view of bioluminescence image (*R*^2^ = 0.80) and **g** to ventral view of bioluminescence image (*R*^2^ = 0.35). ^*^Two data points (2 × 10^8^) are overlapping in **e**

We demonstrated the feasibility of InVivoPLOT on a bioluminescence imaging example by mapping the spatial distribution of CFU of luminescent bacteria inside a live mouse. Using the obtained bacterial density distribution in tissue (CFU mm^−3^) and the OPM as organ ROI template, we were able to directly determine the bacterial organ burden. To calculate the in vivo bacterial burden of the mouse kidney (pyelonephritis), we utilized an established UTI model by inoculating the bladders of C3H/HeJ mice (*n* = 8) with small volumes of uropathogenic *E. coli CFT073* stably expressing the *luxCDABE* operon (lux-bacteria) by transurethral catheterization^39^. The in vivo bacterial burden in the kidneys was determined 24 h after inoculation by employing BLt. The anesthetized and partially shaved mice were placed into a BCAM corresponding to their weight (Fig. 3a–c), the BCAM was loaded into the mirror gantry (Fig. 3e, f), and connected to the gas anesthesia supply. Four spectral images (550–720 nm, 50 nm bandwidth, 300 s), pertaining to the spectral range with largest variation of tissue light absorption, were taken with the PhotonImager (BiospaceLab, France). The EM algorithm reconstructed the photon emission density (photons s^−1^ mm^−3^) of each animal. The unknown bacterial density distribution (CFU mm^−3^) was determined by multiplying the reconstructed photon emission density by a calibration factor. This factor was obtained from a single calibration experiment where the bacterial organ burden and photon emission density were known. The spatial CFU density distribution was coregistered to the OPM and the location of the bacterial infection was instantaneously determined (Fig. 6a–d). Postimaging, CFUs in each of the whole mouse kidneys were quantified by serial plating. The total in vivo CFU count, determined from the BLt reconstructions and the calibration experiment, were plotted versus the ex vivo microbacterial CFU count from serial-plating (Fig. 6e). The Pearson correlation coefficient of the in vivo CFU with respect to the ex vivo CFU was *R*^2^ = 0.93. In contrast, the bioluminescence photon count of images directly taken at the BCAM’s dorsal (Fig. 6f) and ventral (Fig. 6g) surface was plotted versus the ex vivo microbacterial CFU count, yielding Pearson correlation coefficients *R*^2^ = 0.80 and *R*^2^ = 0.35, respectively.

**Fig. 7:**
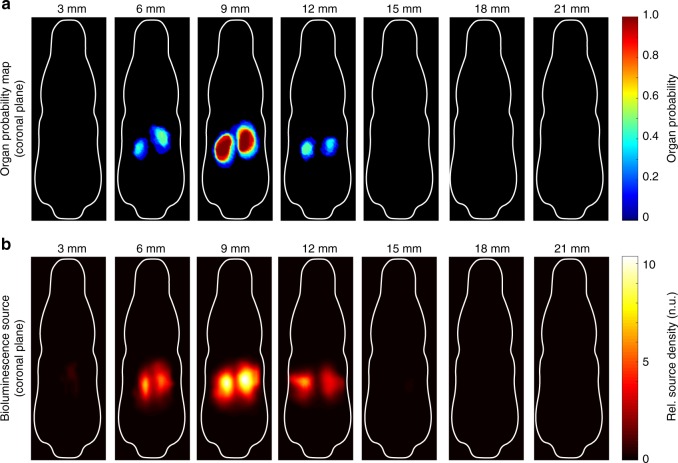
BLt reconstruction of kidney-specific luciferase reporter mouse and alignment with OPM. **a** Coronal slices of kidney OPM confirm luminescence signal emanating from kidneys. **b** Coronal slices of reconstructed luminescence source density of a kidney-specific luciferase reporter mouse *SLC34a1-R26-Luc*. Reconstruction confirms ability to delineate two organs in close proximity (left and right kidneys)

In addition to the UTI experiment with bacterial distributions in the entire urinary tract, the spatial reconstruction accuracy of BLt was validated in a kidney-specific luciferase reporter animal, where the location of the luminescence sources was known to be in the kidneys only. We found that the reconstructed emission density distribution of the kidney-specific luminescence strongly correlated with the location of both kidneys (Fig. 7).

## Discussion

Data analysis of bioluminescence images is based on manually drawn ROIs of luminescence light intensities. The measured intensities depend on different imaging conditions such as imaging view, spatial reporter probe location, and animal pose relative to the camera and, hence, make data quantification challenging (Fig. 1)^51^. The absense of image data congruency across animals with different size and pose further prohibits automated data analysis (Fig. 2). We addressed those challenges by developing InVivoPLOT, which consists of a BCAM, a multiview mirror gantry with gas anesthesia supply and gas scavenging (Fig. 3), an OPM (Fig. 4), and a luminescence source reconstruction algorithm for whole-body BLt. InVivoPLOT unifies the in vivo quantification and automated analysis of bioluminescent reporters in animals across entire animal cohorts. The BCAM plays a key role in that process because (i) it facilitates BLt by providing a defined surface geometry of the animal and (ii) it enables data point congruency between animals.

The known geometry of the BCAM enables the application of BLt without the need for additional surface registration strategies and without repetitive calculations of the EM projection matrix for different animal experiments. The matrix is prebuilt, yielding a BLt reconstruction sped-up by several orders of magnitudes because a light propagation model does not need to be solved for each reconstruction task. Furthermore, the BCAM provides data congruency across animals because each animal assumes the same surface geometry. Therefore, automated data analysis can be achieved by directly comparing corresponding image data points of different animals or time points (Fig. 2).

As part of our feasibility study, we built different BCAM sizes that can fit animals of varying body weights between 17 and 38 g (Supplementary Fig. 1). We validated the goodness of fit of animals having different weights to their corresponding BCAM size by visual inspection of CT scans. We visually examined whether the animal’s skin or fur was in close contact with the BCAM (Supplementary Fig. 2) across a set of BCAM sizes between 17 and 31 g. We did not observe any physiological stress of the anesthetized animal caused by the body-fit enclosure of the BCAM shells throughout a period of at least 60 min. The distance of two BCAM surfaces of adjacent BCAM sizes changes only by approximately 0.1–0.3 mm along the lateral or dorsal–ventral direction and, in comparison, the MSD of most organs of the male C57BL/6 OPM is larger (0.3–0.7 mm, Table 1) than the spatial tolerance of a BCAM for given body weight. This observation also suggests that an incremental step of 1 g between BCAM sizes is sufficient, because the biological variability of organ sizes (MSD, Table 1) between animals of same weight and the variability of repeated animal placement into the BCAM remains to be the limiting factor.

The OPM provides an anatomical reference to the BLt reconstruction and is aligned to the BCAM via a rigid body transform. The OPM functions as an organ ROI template, which enables automated biodistribution analysis without the need for manual delineation of organ ROIs. Currently, the rigid body transform of the OPM can only be applied to mouse models where the anatomy of the diseased state does not significantly deviate from its naïve state corresponding to the OPM. Analysis of animals with an anatomy different from the OPM would either require a nonrigid body transform of the OPM taking anatomical abnormalities into account or require a modification to the surface geometry of the BCAM, for example, allowing imaging of surface protruding tumors, which are all challenges to be addressed in future studies.

The OPM was also utilized for building nonuniform optical property maps that became input to the light propagation model of the BLt reconstruction algorithm. Currently, the optical property maps were calculated by weighted averaging of optical properties of different organs using the OPM probabilities. Alternatively, these maps could be built by using the probability threshold instead, which delineates distinct organ boundaries and optical properties will be assigned to organs without weighted averaging. Future studies still need to be performed for systematically validating different atlas-based optical property maps.

We constructed OPMs of different mouse strains and validated their anatomical registration accuracy by calculating the Dice, VRC, MSD, and dMSD. Best registration accuracies were observed for probability thresholds (*p*) of the OPM between 30% and 50% (*p* = 0.3–0.5) (Fig. 5a–c). Using a 40% probability threshold (*p* = 0.4), we found a Dice of 0.72–0.89 for all major organs (except bladder) for the female C57BL/6 strain, a Dice of 0.53–0.84 for the male C57BL/6 strain, and a Dice of 0.65–0.91 for the C3H strain (Table 1). The Dice coefficients of the male C57BL/6 strain are slightly smaller when compared to the other OPMs, because we were using animals with different body weights (17–31 g) as an expert data set. The smallest Dice (0.53–0.83) were observed for the spleen, which is known to be challenging for atlas registration^32^, and for the skeleton (0.65–0.74). The largest Dice were obtained for the brain (0.84–0.91). Although we did not include the bladder into our registration study, we still calculated its OPM registration metrics. Volume and location of the bladder largely depend on its urine content. Our OPM registration results are also in good agreement with data reported by various groups (Fig. 5e), while yielding excellent registration results for most organs. The generalized MSD yielded similar registration results for each organ and we found mean values between 0.19 mm for the brain and ~0.7 mm for liver and spleen (Fig. 5d). The dMSD has mean values between 0.2 mm for the brain and 0.8 mm for the liver. For most organs, both surface distances are in the same range as the voxel resolution (0.44–0.58 mm) of the scaled OPM. This result suggests that the spatial registration accuracy of individual organs is within the given OPM resolution. Last, we observed that the standard deviations of the Dice coefficient and MSD were significantly larger for the spleen when compared to other organs, indicating that the spleen has a larger spatial location variability across different animals. Smallest standard deviations were found for the brain, indicating the most consistent spatial registration across different animals.

The supporting BLi and BLt experiments of luciferase-expressing kidneys provided evidence that the luminescence light distribution on the tissue surface is not significantly impacted by the optical properties of the BCAM (Supplementary Fig. 3) and that both kidneys in close proximity could be clearly delineated in the reconstructed images of the source density (Fig. 7). The BLi experiment of top shell removed vs top shell closed showed that the luminescence light distribution at the tissue surface is similar for both experimental conditions. The polycarbonate shells uniformly attenuate the exiting light by partial absorption, but do not distort the surface light distribution due to partial reflection along the optical BCAM-to-air interface. We also observed that an animal weight tolerance of ±0.5 g for given BCAM size is acceptable for providing a close skin to BCAM contact and, thus, yielding reproducible BLt reconstruction results.

The in vivo CFU count of kidney tissue was determined by using the reconstructed light emission density and the kidney OPM with given probability threshold of 30% (*p* = 0.3) as organ ROI template (Fig. 6a–d). The CFU density was integrated within the defined OPM boundary yielding the total CFU count of the kidney. The kidney OPM threshold was obtained from the largest Dice coefficient associated with the best OPM registration accuracy. Future studies are still necessary to further explore alternative methods for calculating the total CFU count, for example, by using maximum, mean, or median values within the organ ROI. Last, the total in vivo CFU count was compared to the ex vivo CFU count obtained from serial plating, yielding a Pearson correlation coefficient of 0.93 (Fig. 6e). In comparison, correlation coefficients of only 0.8 and 0.35 depending on the imaging view (dorsal vs. ventral) were obtained with standard BLi analysis (Fig. 6f, g). These results demonstrate that automated ROI analysis using BLt data and a kidney OPM is feasible. More validation studies are still necessary for exploring other organ ROIs and disease models.

**Fig. 8:**
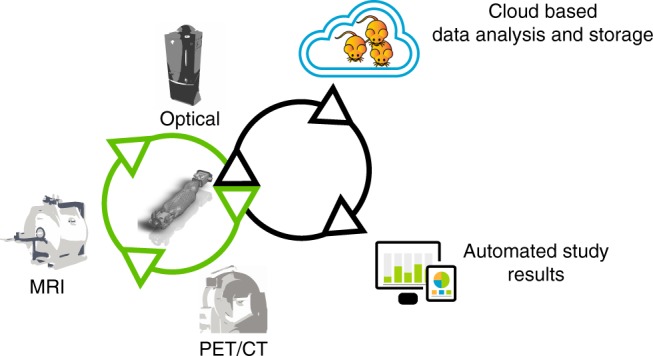
Schematic of cloud-based data analysis platform for multimodal preclinical imaging. The BCAM and the OPM are part of a platform technology, which connects different imaging systems (e.g., optical, PET/CT, and MRI) to enable automated cloud-based data analysis. Collected images are uploaded via a browser-based web interface to a server for data annotation, storage, analysis, and automated study report generation. Automation by this *machine* moves the investigator from in the loop, to on the loop, which will improve accuracy and reproducibility of small animal imaging studies

In this study, we demonstrated the feasibility for determining the in vivo CFU count in kidneys of a urinary tract infection model using InVivoPLOT, which is comprised of the BCAM, a BLt reconstruction algorithm, and the OPM. The BCAM also provides data congruency across different animals, time points, and imaging modalities and, hence, enables automated data analysis of bioluminescence images. The presented OPMs are currently limited mostly to quantifying BLt data in models that do not greatly deform the anatomy such as monitoring infections^39^, T-cell dynamics, in vivo toxicology and pharmacology, gene expression^52^, and early cancer metastases^53^. InVivoPLOT will be extended in the future to other preclinical imaging modalities such as positron emission tomography/CT and magnetic resonance imaging (Fig. 8), and commonly used mouse strains (BALBc, Nude, NSG, and others) will have OPMs generated to increase target identification power. Additional imaging modalities will be tested for both the OPM and coregistration with BLt. In this context we conclude that the BCAM in conjunction with BLt and a statistical mouse atlas facilitates quantitative and automated data analysis of bioluminescence images.

## Methods

### Bioluminescence tomography of lux-bacteria

The female C3H/HeJ mice (24 ± 0.5 g) with pyelonephritis (UTI) were anesthetized, shaved, and placed inside the 24 g BCAM and positioned into the mirror gantry of the plugin unit. Bioluminescence light intensities (photons mm^−2^ s^−1^) were measured at the animal’s tissue surface by taking camera images with a PhotonImager (BiospaceLab, France). Images were taken at four (*Λ* = 4) partially overlapping spectral windows between 550 and 720 nm (550–600, 590–640, 630–680, 670–720 nm), each having a spectral window of 50 nm. Camera integration time was 300 s using the largest available lens aperture (*f*/1.4). Insufficient light intensity levels for performing BLt were observed at wavelengths <550 nm due to the strong light absorption of tissue. The mirror gantry images provided a complete 360° view of the entire animal surface. The light emission kinetics of the lux-bacteria was monitored with open filter images (30 s), which were taken at the beginning, at the end, and in between spectral images, and spectral light intensities were corrected accordingly. In addition, several other light intensity corrections have been performed, which take the partial mirror reflection for single reflection (*R* = 0.9) and double reflection (*R* = 0.81), the exiting angle of photons at the BCAM surface with respect to the camera, the bioluminescence spectrum of lux-bacteria, and filter characteristics into account. The light intensities of the spectral images were then mapped onto the surface of a digital model of the BCAM. The BCAM surface was defined on a 3D structured Cartesian grid with dimensions of 160 × 60 × 46 grid points (length × width × height) and grid point separation of 0.5 mm (regarding the 24 g BCAM) yielding a total of approximately 200,000 grid points or image voxels. Each grid point also constituted an unknown bioluminescence source point (*S*). A subset of equally spaced surface grid points of the BCAM model constituted virtual detector points, yielding approximately a total of *D* = 3600 points for each spectral window. Next, the detector readings were fed into the EM algorithm, which calculated the 3D spatial distribution of the light emission density (photons mm^−3^ s^−1^)^24^. The projection matrix of the EM algorithm had *S* × *D* × *Λ* ~2.88 × 10^9^ elements according to the number of source points (*S*), detector points (*D*), and spectral windows (*Λ*). The matrix elements were calculated by the SP_3_ light propagation model prior to the source reconstruction process and results were digitally stored. The EM algorithm ran on eight Intel Xeon cores (2.7 GHz clock rate) and the source reconstruction was completed in less than 60 s. Last, the reconstructed light emission density was transformed into the bacterial density (CFU mm^−3^) by using a single calibration point, where the light emission density and its actual CFU count (determined by serial plating) were known (Fig. 6).

### BLt of kidney-specific luciferase reporter

*ROSA26 L-S-L-Luc/* *+* *[FVB.129S6(B6)-Gt(ROSA)26Sortm1(Luc)Kael/J, 005125]*^54^ mice were bred with *SLC34a1-CreER(T2)*^55^, and Cre-recombinase expression was activated by tamoxifen administration, to create a conditional kidney-specific firefly luciferase reporter mouse (*SLC34a1-LSL-R26-Luc*). Cre-recombinase was activated by tamoxifen dissolved in filtered corn oil (20 mg/mL) at 37 °C and injected i.p. (100 mg/kg) every other day for 4 days using 8-week-old mice to create a firefly luciferase kidney-specific reporter mouse (*SLC34a1-R26-Luc*). Regarding the BLt experiment (Fig. 7), the animal (26 g) was anesthetized, shaved, and luciferin was injected i.p. (150 mg luciferin/kg body weight). The animal was placed into the 26 g BCAM and mirror gantry, and positioned inside an IVIS Spectrum (PerkinElmer, United States). Four spectral images between 580 and 660 nm (580–600, 600–620, 620–640, 640–660 nm) with nonoverlapping spectral windows of 20 nm were taken with medium lens aperture (*f*/4). The camera integration time was 300 s. Luciferin kinetics was monitored with open filter images, acquired at the beginning, at the end, and in between the spectral images. Similar image intensity corrections were performed as already described above, and bioluminescence source reconstruction was performed using the EM algorithm and its precalculated projection matrix. Reconstructed images of relative light intensities were automatically registered to the OPM. Regarding the BLi experiment (Supplementary Fig. 3), two animals (27 and 29 g) were anesthetized, shaved, luciferin was injected i.p. (150 mg luciferin/kg body weight), and were placed inside the 27 and 29 g BCAM, respectively, and positioned on the imaging stage inside the IVIS Spectrum. To study the impact of the BCAM top shell on the luminescence light distribution, we acquired a sequence of six (*n* = 6) open filter images by alternating the imaging conditions between top shell removed (Supplementary Fig. 3a) and *top shell in place* (Supplementary Fig. 3b). The camera integration time was 180 s using a medium camera aperture (f/4). Images were corrected for luciferin kinetics by normalizing each image to its total light intensity value (total detected photon flux at BCAM surface). A mean image (*n* = 3) was calculated for each of the image sequences top shell removed (Supplementary Fig. 3c) and top shell in place (Supplementary Fig. 3d).

### Construction of the OPM

The female C3H OPM, which was used for the UTI study, was built from contrast-enhanced CT scans using the vascular imaging agent AuroVist 1.9 *nm* (Nanoprobes). AuroVist injection (100 µg) was performed by femoral vein catheter. Each anesthetized C3H mouse (*n* = 11) with average weights of 24 ± 0.5 g was placed inside the 24 g BCAM and onto the imaging bed of a micro-CT system (Bioscan/Mediso, United States). Imaging was performed 60 min postinjection. Nine major organs (skeleton, lung, kidneys, liver, heart, bladder, spleen, brain, and eye) were manually segmented with Mimics (Materialise). The male C57BL/6 OPM was built from contrast-enhanced CT scans of C57BL/6 mice (*n* = 11) using Exitron 12000 (Miltenyi Biotec) by femoral vein catheter (100 µg). Imaging was performed 4 h postinjection using a micro-CT system (Bioscan/Mediso, United States). Nine major organs (skeleton, lung, kidneys, liver, heart, bladder, spleen, brain, and eye) were manually segmented with Mimics (Materialise). The female C57BL/6 OPM was built from contrast-enhanced CT scans using Exitron 12000 (Miltenyi Biotec) by tail vein injection (100 µg). Imaging was performed 60 min postinjection. C57BL/6 mice (*n* = 9) with average weights of 20 ± 0.5 g were placed inside the 20 g BCAM and CT imaging was performed with a high-res Crump CT system (UCLA Crump Institute for Molecular Imaging, United States). Nine major organs (skeleton, lung, kidneys, liver, heart, bladder, spleen, brain, and eye) were manually segmented with Mimics (Materialise). The segmented organs were mapped onto a structured Cartesian grid with 160 × 60 × 46 grid points corresponding the digital BCAM model. The spatial grid point separation is a function of the BCAM size and is, for example, 0.5 mm for the 24 g BCAM and 0.47 mm for the 20 g BCAM. The OPM was generated by determining the probability 0 ≤ *p*_*i*_^*j*^ ≤ 1 at each image voxel *i* for a given organ *j*. The highest (lowest) probability *p*_*i*_^*j*^ of (not) finding a given organ *j* at voxel *i* is *p*_*i*_^*j*^ = 1 (*p*_*i*_^*j*^ = 0). The maximum probability, max (*p*_*i*_^*j*^), of all *j* at given mutual voxel determines a nonoverlapping boundary between different organs. Figure 4c and Supplementary Figs. 4–12 show the C3H OPM for different organs used in the UTI study.

### Validation of the OPM

The C3H and C57BL/6 atlases (designated by *R*_OPM_) were validated with expert data sets (designated by *R*_E_) of segmented CT scans of the same strain and sex. Each OPM organ had a varying organ boundary determined by the user-defined minimum acceptable OPM probability (Fig. 5a–c). Therefore, the anatomical registration accuracy of the OPM was a function of the given probability threshold. The registration accuracy of each atlas was determined by voxel-wise comparing its organ probabilities larger than a given probability threshold to individual segmentations of the expert data set *R*_E_. Dice coefficients, VRC, generalized MSD, and dMSD were calculated for each segmented CT scan, and the mean was determined for the entire CT data set. The Dice coefficient is the registration accuracy for each organ and is given by: $2\frac{{|R_{{\mathrm{OPM}}}\mathop { \cap }\nolimits R_{\mathrm{E}}|}}{{|R_{{\mathrm{OPM}}}| + |R_{\mathrm{E}}|}}$^32^. The VRC is the ratio $\frac{{|R_{{\mathrm{OPM}}}|}}{{|R_{\mathrm{E}}|}}$ of the recovered organ volume of data sets *R*_OPM_ and *R*_E_. The generalized MSD is the bidirectional mean surface distance or average surface distance^36^ between the organ surfaces of the OPM and the segmented CT scan of the expert data set: $\frac{1}{2}\left( {\frac{1}{{N_{{\mathrm{OPM}}}}}\mathop {\sum }\nolimits_{i = 0}^{N_{{\mathrm{OPM}}}} d_i + \frac{1}{{N_{\mathrm E}}}\mathop {\sum }\nolimits_{j = 0}^{N_{\mathrm{E}}} d_j} \right)$. *N*_OPM_ and *N*_E_ are the numbers of surface points of the OPM and the organ-segmented CT scan. The distance *d*_*i*_ indicates the minimum distance of the *i*-th surface point of the OPM organ with respect to all surface points of the organ of the expert data set. The distance *d*_*j*_ constitutes the minimum distance of the *j-*th organ surface point of the expert data set to all points of the OPM organ surface. In contrast, the dMSD is a directional distance^42^ using only distances *d*_*i*_: $\frac{1}{{N_{{\mathrm{OPM}}}}}\mathop {\sum }\nolimits_{i = 0}^{N_{{\mathrm{OPM}}}} d_i$. VRC and Dice coefficients ≈1 indicate an exact match, whereas the smallest MSD or dMSD [mm] depicts the best registration accuracy. Results were also compared to the outcome of similar anatomical registration studies as shown in Fig. 5d, e. A complete list of all Dice coefficients, VRC, MSD, and dMSD can be found in Table 1.

### Optical property maps

The OPM was translated into spatially nonuniform maps of the tissue absorption, $\mu _a^{}$, and reduced scattering, $\mu _s^\prime (\lambda )$, coefficients to address the different optical properties of organs. The optical properties are needed for the light propagation model of the BLt image reconstruction. The spectrally dependent maps of $\mu _a^{}(\lambda )$ and $\mu _s^\prime (\lambda )$ were built for four (*λ* = {1,2,3,4}) partially overlapping wavelength intervals of 50 nm between 550 and 720 nm, which pertains to the spectral range with largest variation of tissue light absorption. Each voxel element *i* of the spectral maps at interval *λ* constituted the expectation value $\bar \mu _i(\lambda )$, which is composed of the organ probabilities $p_{{i}}^{{j}}$ and optical properties $\mu _i^j(\lambda )$ of organ *j*. It is defined as $\bar \mu _i(\lambda ) = \mathop {\sum }\nolimits_{j = 1}^J p_i^j\mu _i^j(\lambda )$ with $\mathop {\sum }\nolimits_{j = 1}^J p_{}^j = 1$. The optical properties $\mu _i^j(\lambda )$, i.e., the absorption and reduced scattering coefficients, were defined by the blood oxygenation level and by Mie-Scattering theory^56–58^. The intermittent or background tissue that was not defined by an organ of the OPM was set to a 80:20 mixture of muscle and fat based on an average tissue fat content of 22.9% for female mice of 40 strains^41^.

### Urinary tract infection

A uropathogenic *E. coli* strain *CFT073* that stably express the *luxCDABE* operon (lux-bacteria), a bioluminescent construct that encodes luciferase and the enzymes required for production of the substrate tetradecanal, by transposon mutagenesis, was previously generated^39^. Female C3H/HeJ (24 ± 0.5 g) were anesthetized with isoflurane 1–1.5%, bodies were shaved, and the lux-bacteria (20 μl of 5 × 10^8^ CFU ml^−1^) was instilled into the bladder through a soft polyethylene catheter (Intramedic, 0.61 mm outer diameter)^39^. The catheters were lubricated using a sterile lubricant gel to reduce discomfort to animals during transurethral catheterization. Immediately after BLi images were acquired, mice were euthanized and kidneys excised to quantify bacterial CFUs. CFUs in kidney homogenates were quantified by serial dilution on LB agar plates.

### Validation of total in vivo CFU count in kidneys

The total CFU count of each kidney was automatically obtained from the reconstructed spatial light emission density distribution (photons mm^−3^ s^−1^) within its kidney OPM boundaries. The kidney boundaries were defined as the 30% probability threshold of the female C3H OPM. First, the CFU density (CFU mm^−3^) at each voxel was determined by using a calibration factor, i.e., each emission density was multiplied by the calibration factor yielding the CFU density. The calibration factor was obtained from an imaging experiment where the emission density within an ROI and its ex vivo CFU count were both known. Last, the CFU density was integrated within the kidney OPM boundaries yielding the total in vivo CFU count.

### Injection of AuroVist and Exitron 12000

Mice were anesthetized with isoflurane and injected through a femoral vein catheter with the recommended dose of the CT contrast agent (AuroVist or Exitron 12000) and kept lightly anesthetized with isoflurane (1–1.5%) for approximately 1 h.

### Animal welfare

The animal experimental protocols described here have been approved by the Institutional Animal Care and Use Committee (IACUC) at the University of Washington, Columbia University, and the University of California Los Angeles (Crump Institute for Molecular Imaging) under strict accordance with institutional and international guidelines and regulations for the use of vertebrate animals.

### Mathematical calculations and MATLAB scripts

Calculations for obtaining the Dice, VRC, MSD, and dMSD were performed with Matlab (MathWorks Inc.).

### Statistical analysis

Matlab (MathWorks Inc.) and GraphPad Prism software (GraphPad Software Inc.) were used for statistical analyses and drawing graphs.

## Electronic supplementary material

Supplementary Information

Description of Additional Supplementary Files

Supplementary Movie 1

Supplementary Movie 2

## Data Availability

The data that support the findings of this study are available from the corresponding authors upon reasonable request.
