# Supplementary material for: Automated quantification of bioluminescence images
Source: Nat Commun. 2018 Oct 15;9:4262. doi: 10.1038/s41467-018-06288-w (PMC6189049; doi:10.1038/s41467-018-06288-w)
Supplement: Supplementary file 1 — Supplementary Information [file 41467_2018_6288_MOESM1_ESM.pdf]

Supplemental Information

Automated Quantification of Bioluminescence Images

Klose et al.

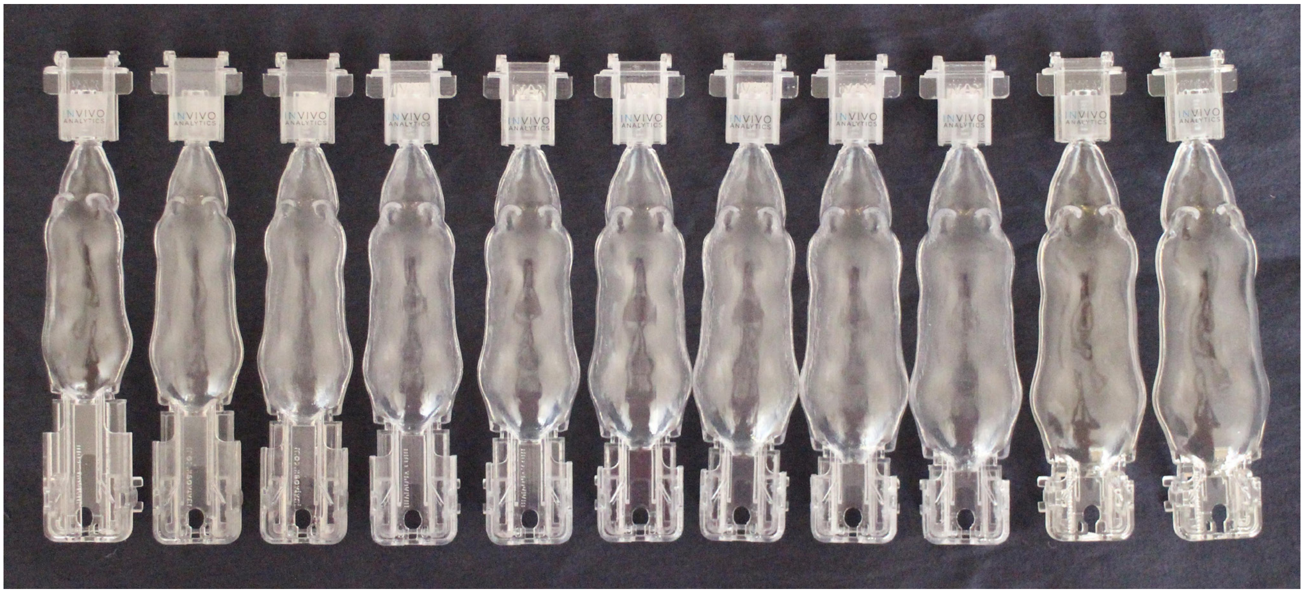

17 g 19 g 21 g 23 g 25 g 27 g 29 g 31 g 33 g 35 g 37 g

Supplementary Figure 1. Current range of BCAM sizes. 17-37g BCAMs in 2g increments displayed.

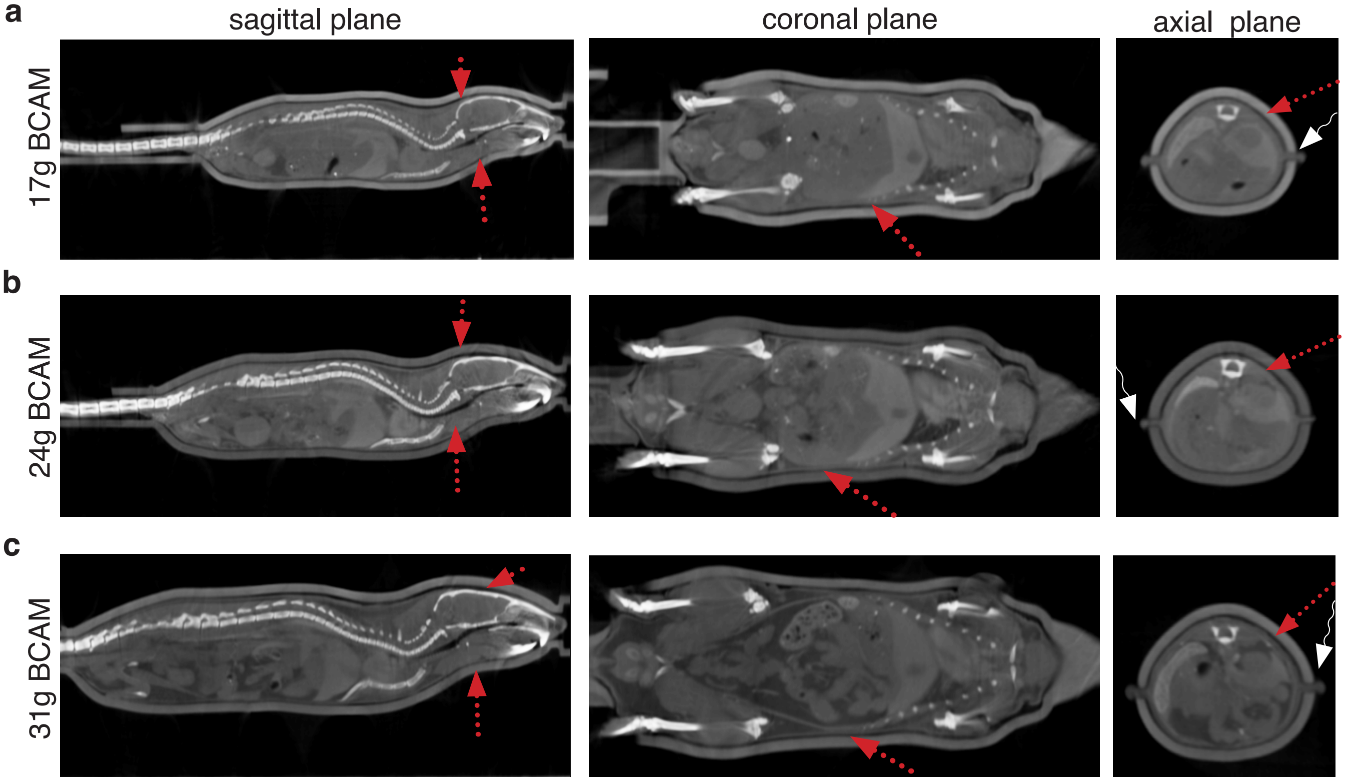

Supplementary Figure 2. Goodness of fit validated by cross-sectional CT images of mouse in BCAM. Goodness of fit is driven by the spatial deviation of the animal along all three spatial axes. Representative sagittal, coronal, and axial CT slices (left to right) from (**a**) 16.8g, (**b**) 24.0g, and (**c**) 30.9g C57BL/6 mice are acquired in 17g, 24g and 31g sized molds, respectively. Some abdominal and head areas show only little space (0.2mm – 0.4mm) between the animal surface (fur) and the inner BCAM surface (indicated by red arrow with dotted line). A lateral gap between the top and bottom shell of the BCAM provides enough space for excess skin and fur to protrude from BCAM volume and helps to maintain a snug fit (white arrow with

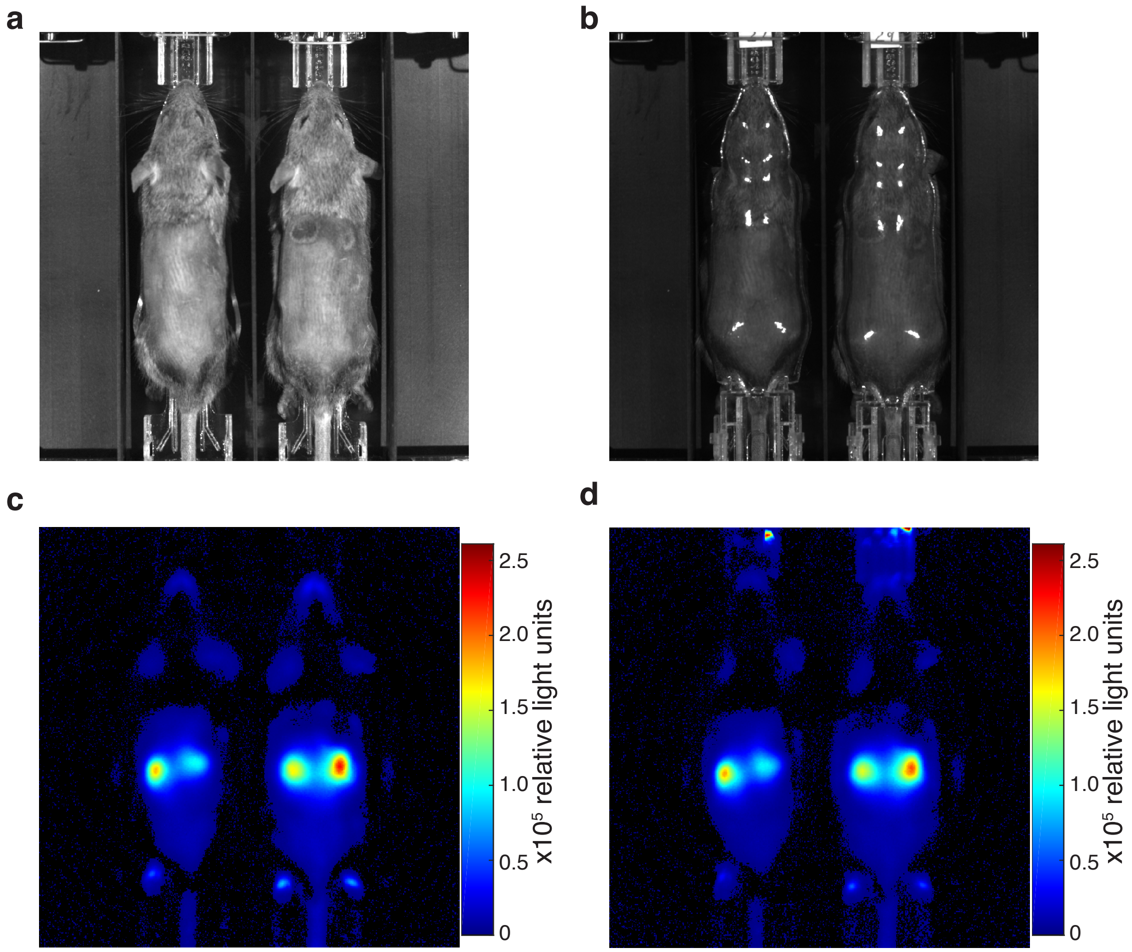

Supplementary Figure 3. Comparison of surface light intensity distribution with and without BCAM. Two SLC34a1–R26-Luc kidney reporter mice (27g and 29g) were placed in a 27g and 29g sized BCAM, respectively. Two imaging experiments were performed: (**a**) top shell of both BCAMs being removed and (**b**) top shell of BCAMs in place. A set of three bioluminescence images (dorsal view) were acquired (**c**) without top shell of BCAM and (**d**) with top shell of BCAM. The bioluminescence images were corrected for the luciferin kinetics and an image of the mean light intensity was calculated. The spatial light distribution of both experimental conditions (‘no top shell’ vs ‘with top shell’) do not show any significant differences while indicating little impact of the BCAM (partial reflection of light due to refractive index mismatch at BCAM-to-air surface, light attenuation due to BCAM material) on the light propagation inside the animal.

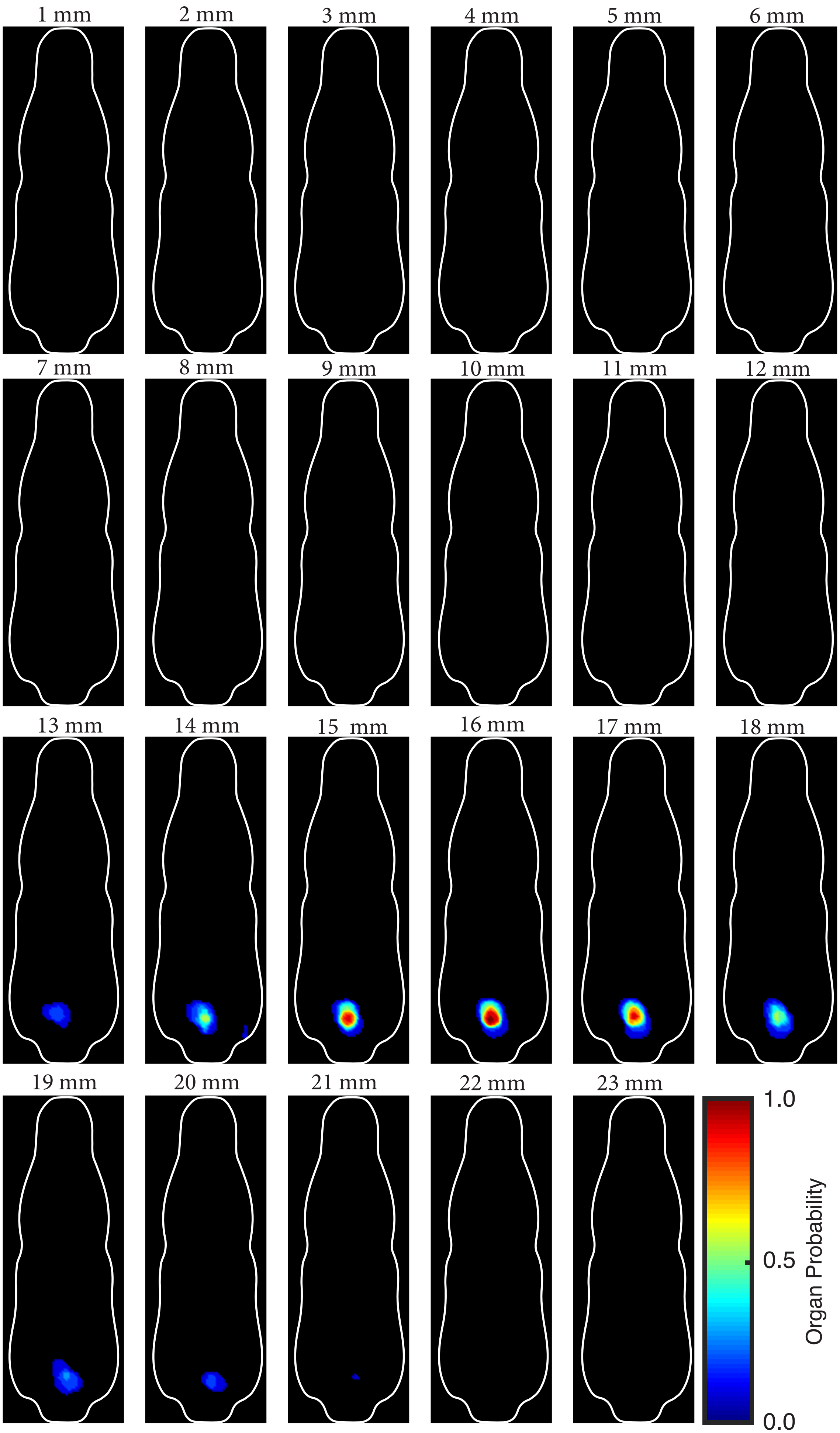

Supplementary Figure 4. Bladder OPM. Coronal sections of bladder from dorsal side to ventral side every 1 mm.

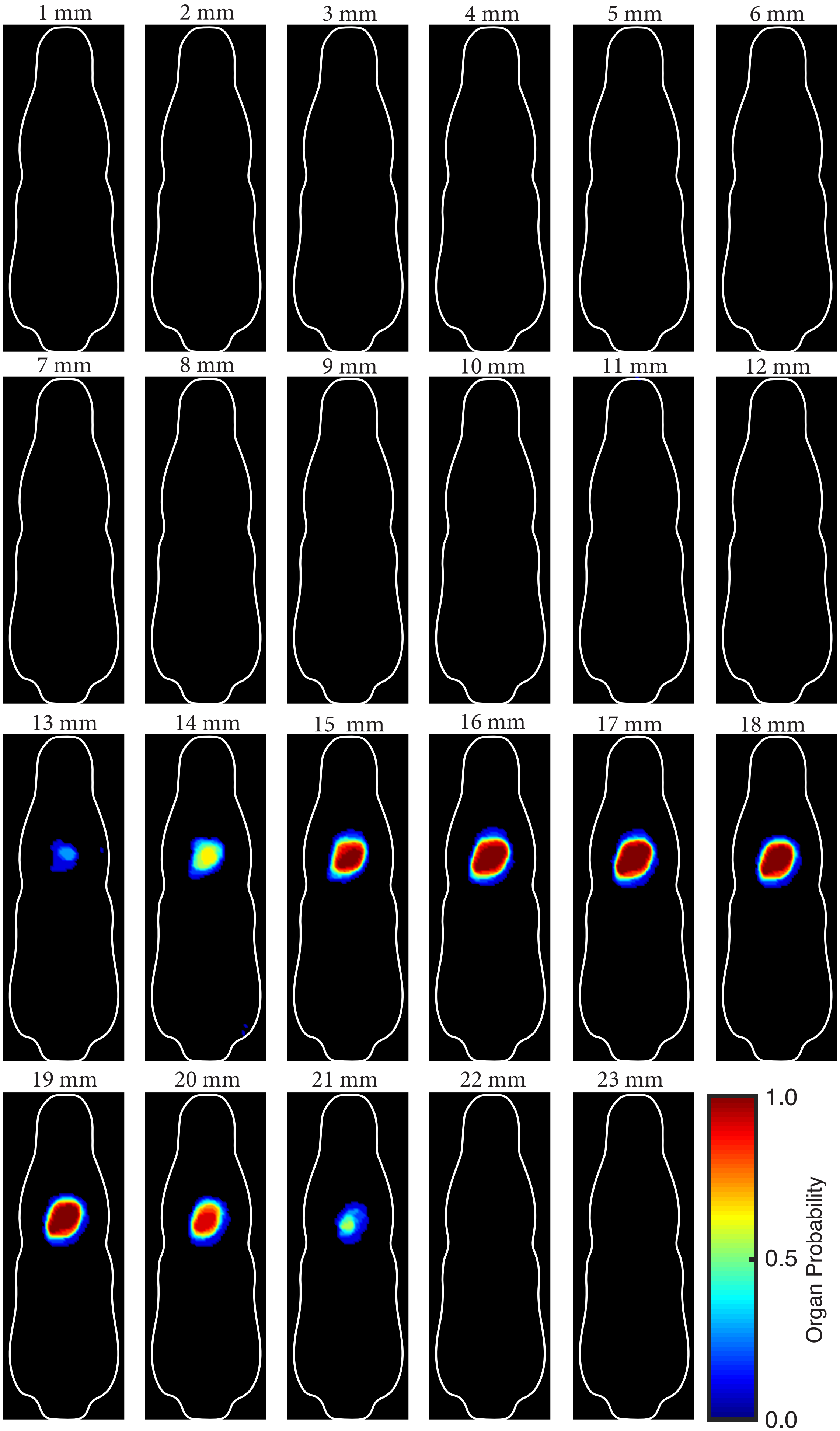

Supplementary Figure 5. Heart OPM. Coronal sections of heart from dorsal side to ventral side every 1 mm.

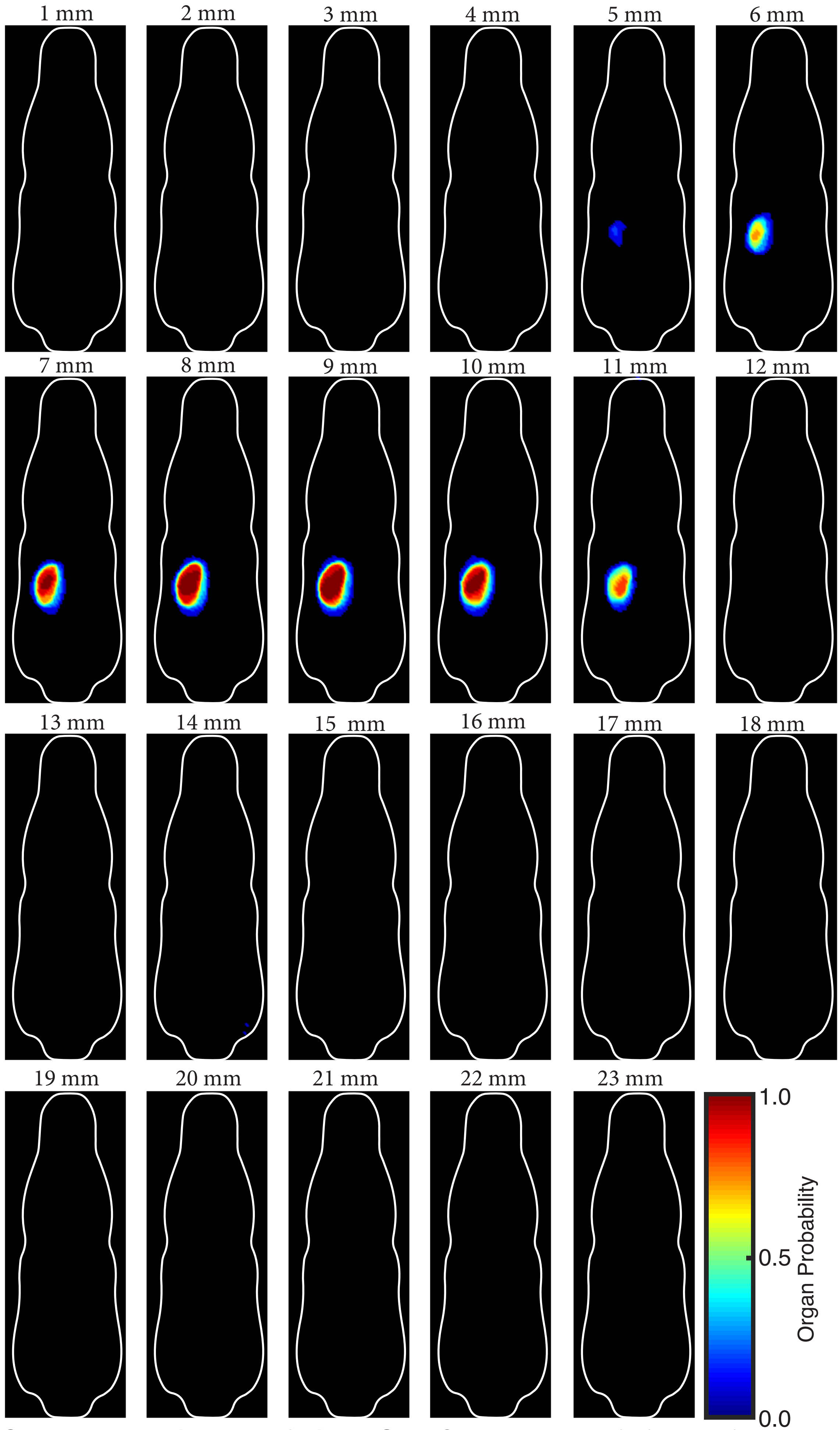

Supplementary Figure 6. Left kidney OPM. Coronal sections of left kidney from dorsal side to ventral side every 1 mm.

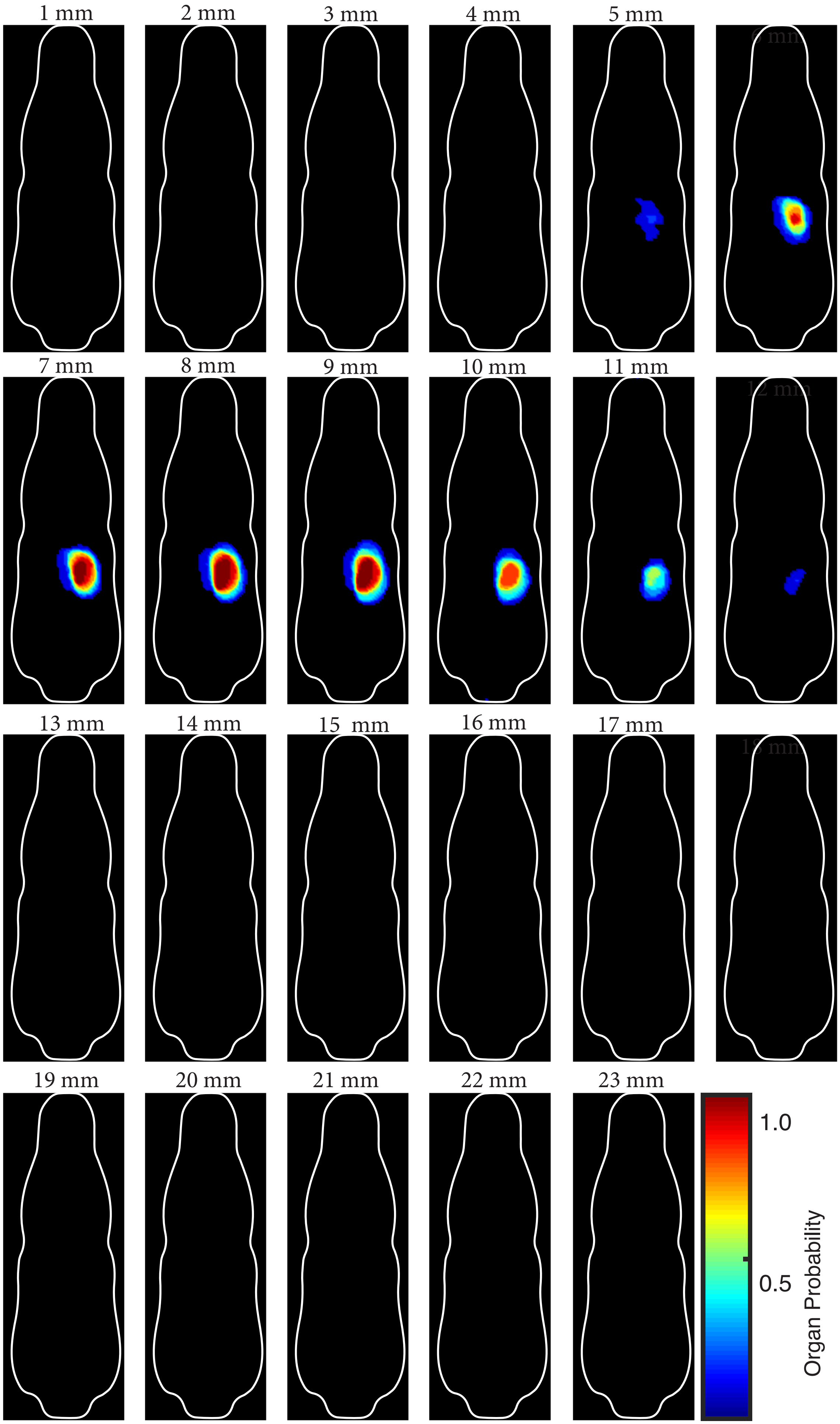

Supplementary Figure 7. Right kidney OPM. Coronal sections of right kidney from dorsal side to ventral side every 1 mm.

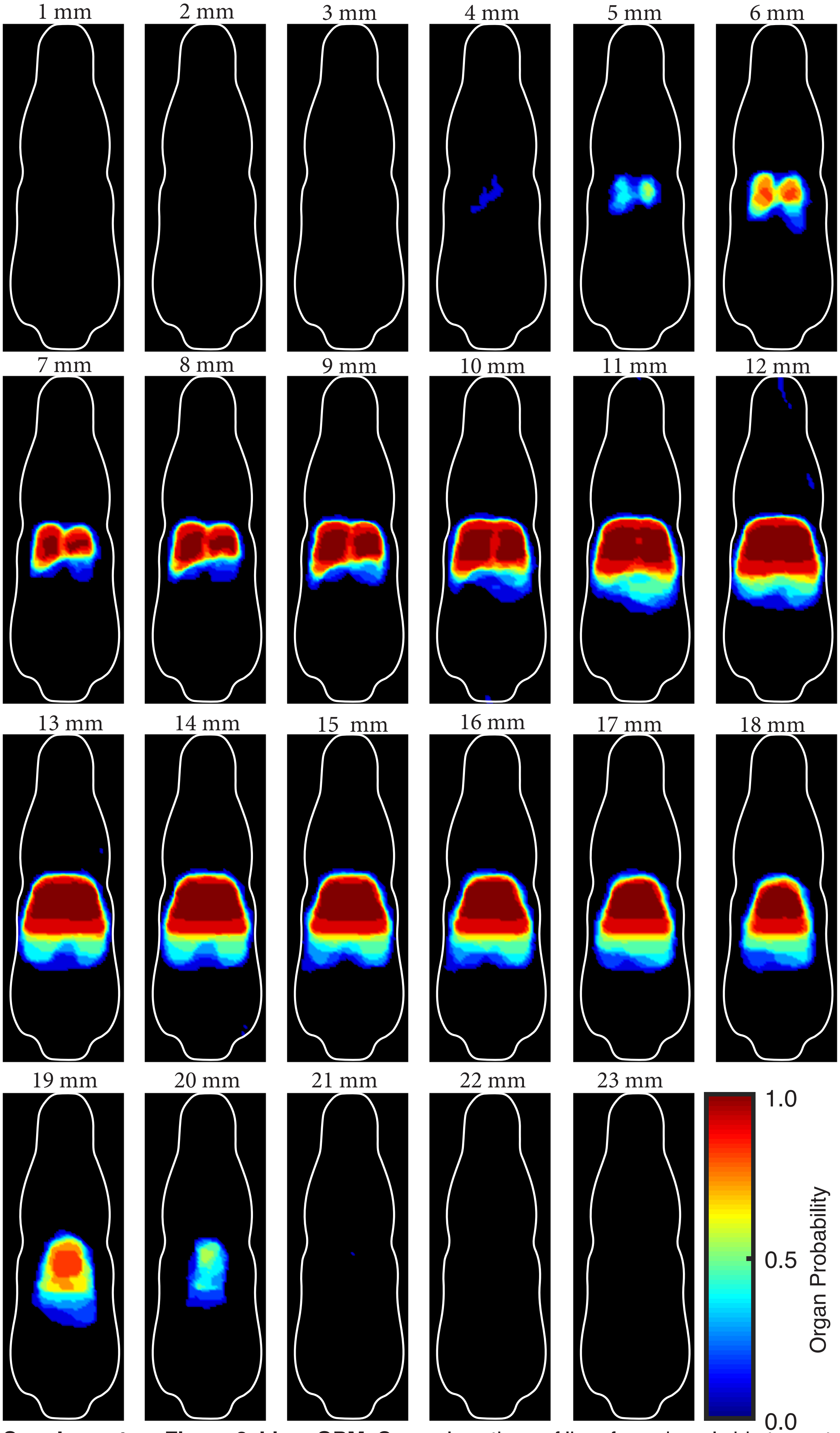

Supplementary Figure 8. Liver OPM. Coronal sections of liver from dorsal side to ventral side every 1 mm.

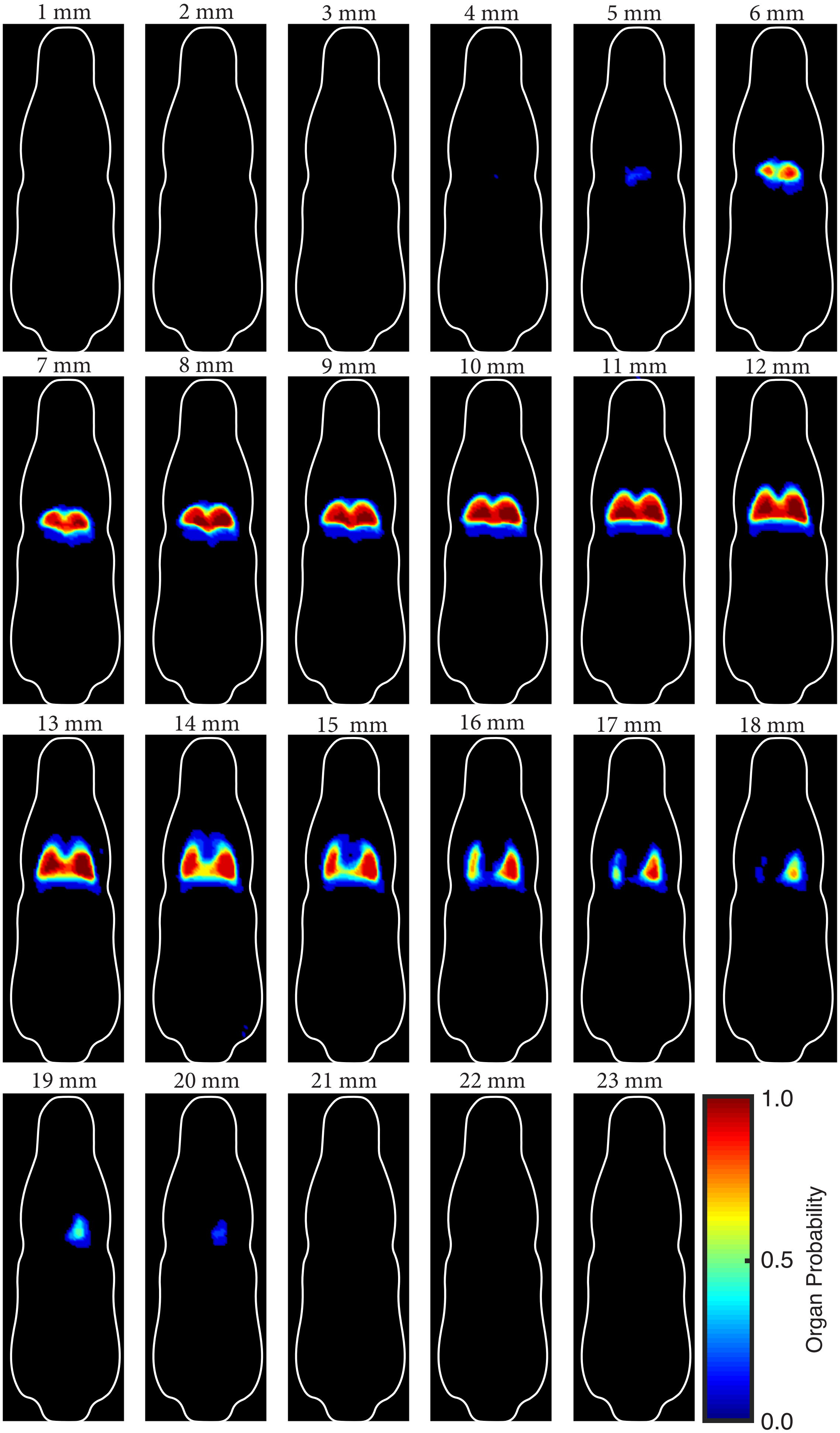

Supplementary Figure 9. Lung OPM. Coronal sections of lung from dorsal side to ventral side every 1 mm.

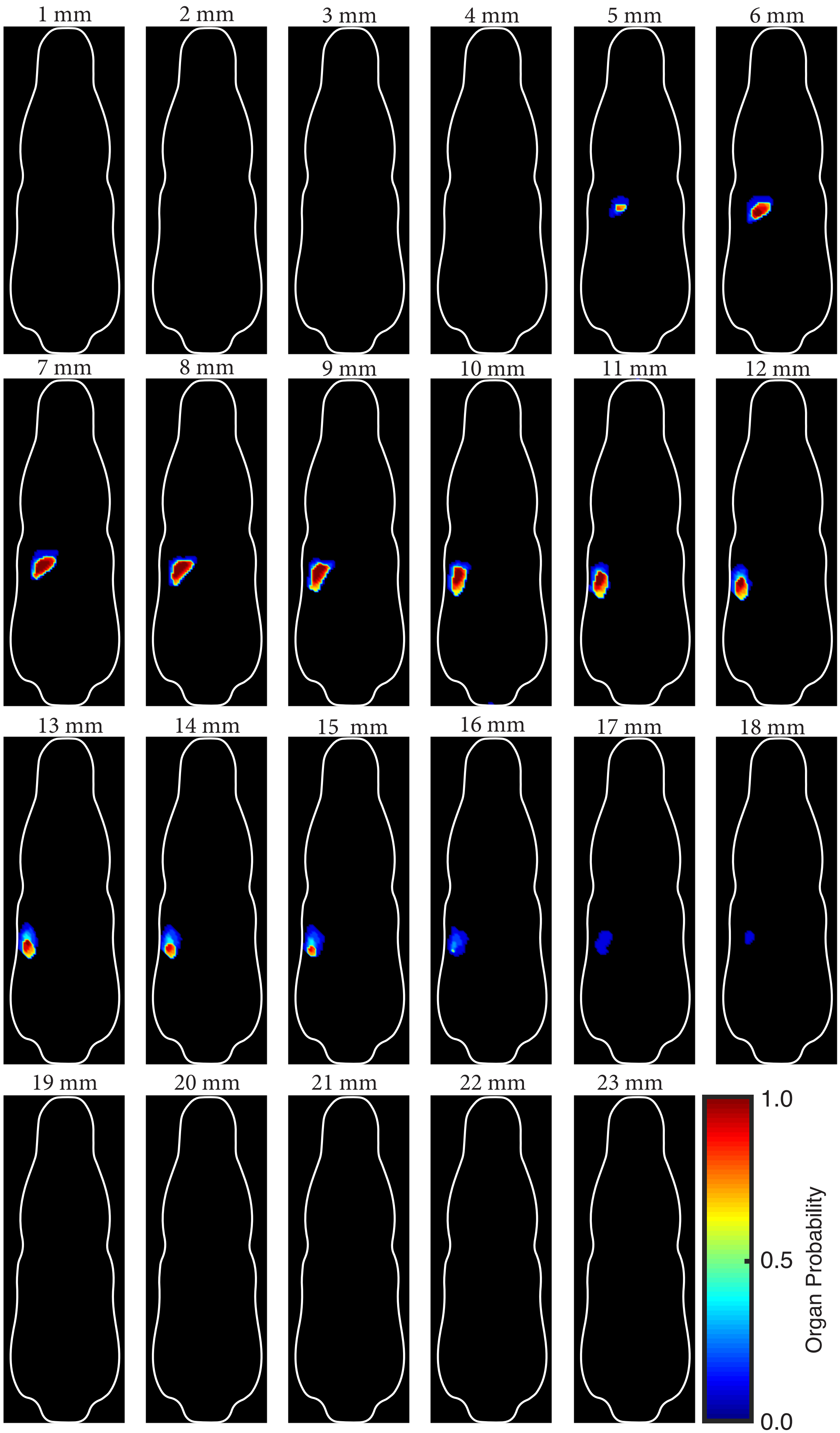

Supplementary Figure 10. Spleen OPM. Coronal sections of spleen from dorsal side to ventral side every 1 mm.

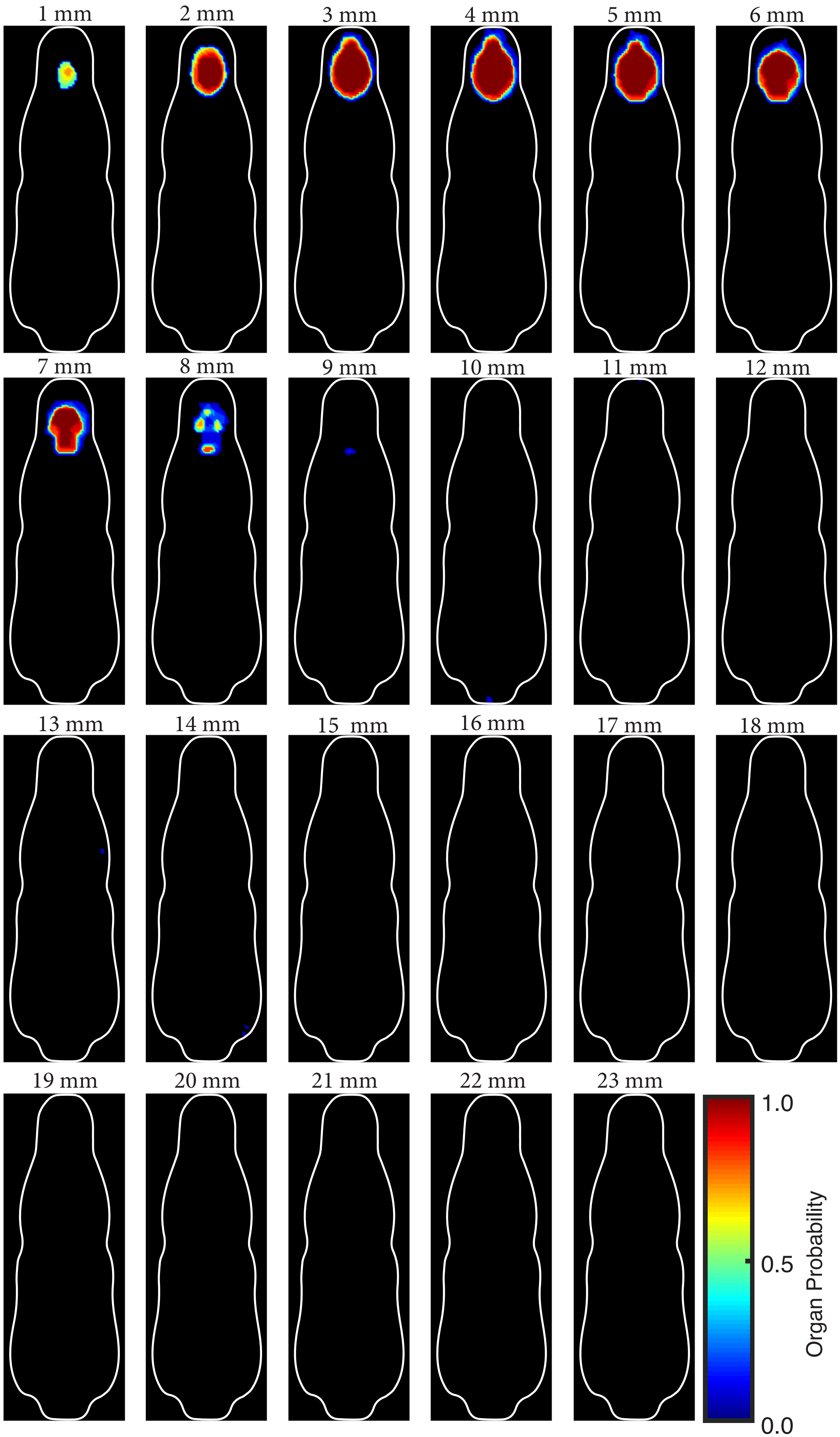

Supplementary Figure 11. Brain OPM. Coronal sections of brain from dorsal side to ventral side every 1 mm.

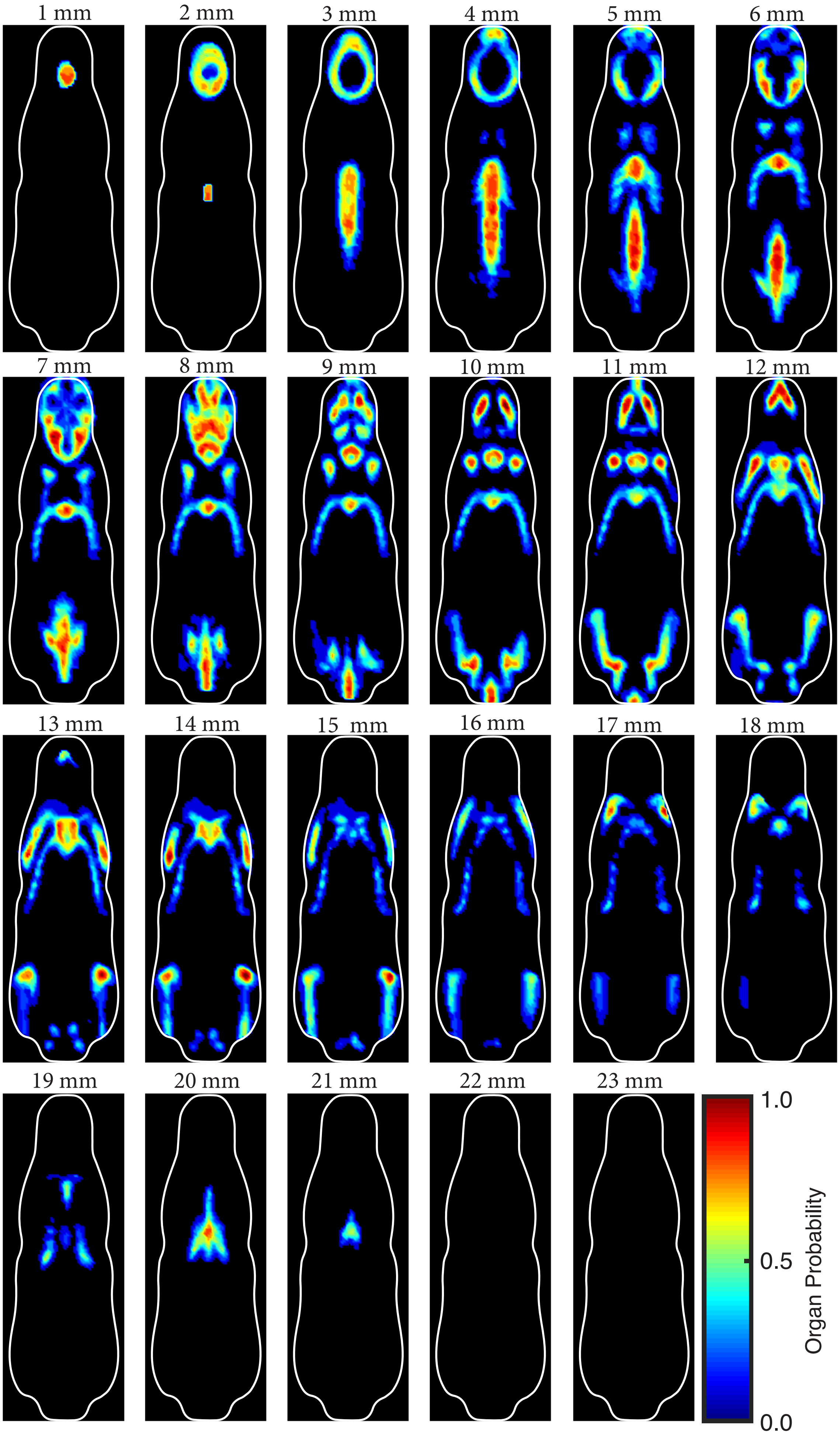

Supplementary Figure 12. Skeleton OPM. Coronal sections of skeleton from dorsal side to ventral side every 1 mm.
