# Supplementary material for: Automated quantification of bioluminescence images
Source: Nat Commun. 2018 Oct 15;9:4262. doi: 10.1038/s41467-018-06288-w (PMC6189049; doi:10.1038/s41467-018-06288-w)
Supplement: Supplementary file 2 — Description of Additional Supplementary Files [file 41467_2018_6288_MOESM2_ESM.pdf]

Description of Additional Supplementary Files

a) File name: Supplementary Movie 1
Description: Open and close of BCAM.

b) File name: Supplementary Movie 2
Description: Skeleton (grey), brain (green), right kidney (yellow), left kidney (yellow), bladder (yellow), lung (blue), liver (cyan), heart (magenta), and spleen (red).
